# The role of dna hypermethylation and demethylation in cancer and cancer therapy

**DOI:** 10.3747/co.v15i2.210

**Published:** 2008-04

**Authors:** M. Szyf

**Affiliations:** Richard J. Ablin, phd, Research Professor of Immunobiology, University of Arizona College of Medicine and the Arizona Cancer Center, Tucson, Arizona, U.S.A., and Phil Gold, phdmd, Professor of Medicine, Physiology, and Oncology, McGill University, Montreal, Quebec, Canada, Section Editors

Methylation of dna is known to be an important mechanism of gene regulation. A hallmark of cancer is the deregulation of the dna methylation machinery and aberrant dna methylation patterns 1. In vertebrate genomes, a large fraction of the CG dinucleotide sequence is modified by methylation in gene-and tissue-specific patterns 2. Methylation of critical regulatory regions silences gene expression; loss of methylation is associated with gene activation 3. Because cancer progression requires many changes in the normal program of gene expression, it stands to reason that aberrations in dna methylation play a critical role in the changes in gene expression involved in cancer progression and metastasis.

Methylation changes in dna play a role very similar to that of genetic mutations in cancer; however, unlike a genetic alteration, dna methylation is potentially reversible with pharmacologic intervention. The dna methylation machinery was therefore proposed— almost a decade and a half ago—to be an attractive anticancer target 4.

## BACKGROUND

Three principal kinds of aberration in the dna methylation machinery occur in cancer:

- Hypermethylation of tumour suppressor genes 5–7
- Aberrant expression of dna (cytosine-5-)-methyl-transferase 1 (dnmt1) and other dnmts that methylate genomic dna involved in processes of gene inactivation, chromatin organization, X chromosome inactivation, and genomic imprinting
- Hypomethylation of unique genes and repetitive sequences

These aberrations in dna methylation have very important diagnostic significance, and using current whole-genome techniques, the methylation signatures of specific cancer types are being delineated. These signatures will play an increasingly important role in diagnosis and prognosis of all cancers 8.

The expression of dnmt1 is tightly regulated with the state of cell growth by transcriptional and posttranscriptional mechanisms 9,10. Several oncogenic pathways lead to overexpression of *DNMT1* 11. Overexpression of *DNMT1* in non-transformed cells causes cellular transformation, which supports the idea that inhibition of dnmt1 would block tumour growth 12. The anticancer effects of dnmt1 inhibition were demonstrated both pharmacologically, using antisense oligonucleotide inhibitors 13,14, and genetically, using *dnmt1*–/– mice 15.

## RATIONALE

To properly design and apply therapeutic strategies that involve dnmt1 inhibition, it is essential to understand why dnmt1 transforms cells and why dnmt1 inhibition blocks tumour growth. The commonly accepted and attractively straightforward model is that dnmt1 inhibition causes loss of methylation during dna synthesis and therefore activation of tumour-suppressor genes that are silenced by dna methylation. Activation of tumour-suppressor genes by demethylation would be expected to block tumour growth. Another possible utility of demethylation agents is demethylation of tumour antigen promoters, which can lead to reactivation of the expression of those promoters, thus increasing the sensitivity of tumour cells to immunosurveillance.

If demethylation is the only mechanism of action of dnmt1 inhibitors, then the goal should be to develop catalytic inhibitors of the dna methylation reaction that would cause global demethylation of dna. However, there are several reasons to question whether dnmt1 inhibition blocks tumour growth exclusively through inhibition of dna methylation:

- Knockdown of dnmt1 by small interfering rna (sirna) or antisense oligonucleotides blocks the growth of cancer cells by mechanisms independent of dna methylation through induction of tumour-suppressor genes 16, triggering a dna damage response and inhibition of dna replication 17.
- The multifunctional protein dnmt1 has multiple domains. To be able to target the clinically relevant actions of dnmt1, delineating the functional domains of dnmt1 that are critical for cancer growth will be important 18.

## APPLICATIONS

Several clinical trials with a nucleoside analogue pan-dnmt inhibitor, 5-azacytidine (5-azaC), and its deoxy analogue, 5-deoxycytidine (5-azaCdR), a potent and non-selective dna methylation inhibitor, have been launched 19. In several clinical trials involving hematologic malignancies, especially myelodysplastic syndrome, several reports showed responses to 5-azaC with tolerable adverse effects 20. However, no significant success has been reported with solid tumours 21. The weak response of solid tumours might be the result of pharmacokinetic issues: delivery problems, or dosing and scheduling problems, or both. Various combination strategies involving 5-azaC and other chemotherapeutic agents, and other chromatin modifiers such as histone deacetylase inhibitors are now being tried 22.

Although 5-azaC has been tested in clinical and preclinical settings for decades, basic questions remain regarding its mechanism of action. It is unclear whether the clinical activity of 5-azaC requires demethylation of candidate tumour-suppressor genes; whether the anticancer effect is a result of activities independent of dna methylation, mediated through 5-azaC binding of dnmt1 and other dnmts; or whether the main activity of this agent relates to its non-specific toxicity as a nucleoside analogue. Similarly, the specific dnmt1 isoforms that are responsible for the anticancer activity of 5-azaC remain undefined.

These unresolved issues have implications for the dosing and scheduling of 5-azaC and for the development of new, more potent dnmt inhibitors. A major issue is the possibility that non-selective demethylation would induce pro-metastatic genes.

Recent trials have focused on low-dose 5-azaC (well below the maximum tolerated dose), under the supposition that 5-azaC causes demethylation at low doses but is mainly toxic at high doses 20. Those trials showed better responses; however, no immediate correlation was observed between the response past a given threshold and the extent of demethylation. Moreover, response was not correlated with the presence of a hypermethylated *p15* before treatment 20.

Another nucleoside analogue that has recently been introduced to the arsenal of dnmt inhibitors is zebularine, a nucleoside analogue, which, unlike 5-azaC, is chemically stable and orally bioavailable. Zebularine was originally identified as a cytidine deaminase inhibitor 23. The compound exhibits dna demethylation activity with reduced potency and toxicity as compared with 5-azaC. Nevertheless zebularine belongs to the same class of nucleoside analogues, raising similar problems to those seen with 5-azaC.

It is unfortunate that the only drug targeting dnmt1 in the clinic is an “old” nucleoside analogue that must be incorporated into dna to perform its action. Thus, although the goal of dna methylation therapy is to target the cell’s machinery in a way that is fundamentally different from classical chemotherapy and thus anticipated to exhibit limited toxicity, the use of a classical nucleoside analogue seems to defeat that purpose.

The many toxicities of 5-azaC result from its properties as a nucleoside analogue and might perhaps mask its activity on dnmts. The only non-nucleoside, isotypic, specific dnmt1 inhibitor that has undergone clinical trial is MG98, a second-generation antisense oligonucleotide that specifically targets dnmt1 messenger rna 24. The mechanism of action of this latter class of inhibitors is different in many respects from that of the nucleoside-analogue catalytic inhibitors of dnmt1. With MG98, the expression of the dnmt1 protein is entirely eliminated, and thus all functional activities of dnmt1 are targeted, including methylation-independent activities. Knockdown of dnmt1 results in inhibition of dna replication 25, triggering a damage response 17 and inducing tumour-suppressor genes 16. The immediate blocking of replication by dnmt1 knockdown dramatically limits the demethylation induced by dnmt1 inhibition, thus avoiding the potential deleterious impact of global demethylation 17.

The chief remaining issue with antisense oligonucleotides is their delivery to solid tumours. The clinical trials of this promising class of drugs were recently stopped because of a lack of objective response. Nevertheless, the overall strategy—and therapeutic sirnas— carries great promise. Searching for agents that knock down dnmt1 rather than inhibit its catalytic activity is a priority that should be pursued.

## CONCERNS AND IMPLICATIONS

Although the principal attention in the field of cancer has been directed at the phenomenon of hypermethylation, a hallmark of the methylation pattern in many tumours is hypomethylation 26. Recent data suggest that demethylation activates metastatic genes such as heparanase 27 and urokinase plasminogen activator, and thus plays an important role in metastasis 28. That finding raises two important questions with critical therapeutic implications:

- First, catalytic inhibitors of dnmts (such as 5-azaC) that cause global hypomethylation and that are now used in anticancer therapy, might increase the propensity of cancer cells to metastasize.
- Second, might demethylation inhibitors be a new approach to cancer therapy? It is therefore critical to develop dnmt1 inhibitors that do not cause demethylation of metastatic genes. A new goal in dna methylation therapy should be the development of dna demethylation inhibitors 26.

Two different approaches were recently used to block demethylation in cancer. The first approach involved treatment with the methyl donor *S-*adenosylmethionine (sam). *S-*Adenosylmethionine contributes the methyl moieties to the methylation reactions and has also been shown to inhibit demethylase activity *in vitro* and in cells 29. Previously, sam was shown to be chemoprotectant in a liver cancer model in rodents 30. Treatment of human breast and prostate cancer cell lines *in vitro* with sam resulted in inhibition of invasion *in vitro,* and metastasis and tumour growth when the cells were transplanted into nude mice *in vivo.* The sam molecule is notoriously unstable 28,31. The results with this agent call for an effort to develop sam analogues with improved pharmacokinetics.

Another important line of investigation involves identifying the proteins responsible for demethylation of metastatic genes in cancer and targeting them for inhibition. One protein—methylated domain dna binding 2 (Mbd2)—was previously suggested to be involved in silencing methylated genes and demethylation alike 29,32. Blocking Mbd2 in breast and prostate cancer cell lines inhibits tumour growth, invasiveness, and metastasis *in vivo* 28,31. The *MBD2* antisense oligonucleotides, *MBD2* sirna inhibitors, and the Mbd2 antagonists are therefore potentially promising antimetastasis candidates.

## SUMMARY

The machinery of dna methylation and demethy-lation represents an attractive therapeutic target; however, certain questions need to be answered before the full potential of this approach is realized. The dna methylation inhibitor currently in use is crude and, by demethylation, could unleash pro-metastasis genes that might increase metastasis. The specific functions of dnmt1 that are involved in tumorigenesis must be isolated from the functions involved in metastasis. Not only dnmts, but also the dna demethylation machinery, are emerging as new targets for inhibition of metastasis—one of the most intractable facets of cancer. The challenge is to design and target the various compartments of the dna methylation machinery to achieve both growth-control induction of tumour antigens and inhibition of metastasis in the absence of adverse effects on methylation.
